# Brainstem Hemorrhage Following Lumbar Drain for Post-traumatic Hydrocephalus

**DOI:** 10.7759/cureus.26349

**Published:** 2022-06-26

**Authors:** Matthew T Carr, Jeffrey Gilligan, Zachary L Hickman, Salazar A Jones

**Affiliations:** 1 Neurosurgery, Mount Sinai Hospital, New York, USA; 2 Neurosurgery, New York City (NYC) Health + Hospitals/Elmhurst, Queens, USA

**Keywords:** decompressive craniectomy, post-traumatic hydrocephalus, case report, brainstem hemorrhage, traumatic brain injury, lumbar drain

## Abstract

Post-traumatic hydrocephalus is common after traumatic brain injury (TBI), particularly following decompressive craniectomy. Cerebrospinal fluid (CSF) removal by lumbar drain (LD) aids in the workup of post-traumatic hydrocephalus and serves as a bridge to definitive CSF diversion. Hemorrhagic complications following LD are rare but can include intracranial hemorrhage. We present a case of fatal brainstem hemorrhage following LD in a patient three months after craniectomy.

A 32-year-old male presented with severe TBI and an acute subdural hematoma. He underwent emergent decompressive craniectomy and hematoma evacuation. The next day, he required ventriculostomy for elevated intracranial pressure (ICP), which was able to be successfully removed. Three months after the injury, the patient’s neurological exam declined, and computed tomography (CT) findings were consistent with communicating hydrocephalus. An LD was placed with 15 mL of CSF and drained every two hours. Five days after LD placement, the CSF became blood-tinged, and a repeat head CT demonstrated an acute brainstem hemorrhage. The patient ultimately expired.

Given the prevalence of post-traumatic hydrocephalus and the frequent use of CSF diversion in the management of this condition, it is important for neurosurgeons to remain cognizant of the potential risk for catastrophic brainstem hemorrhage following LD in decompressive craniectomy patients.

## Introduction

Post-traumatic hydrocephalus is a well-recognized sequela of traumatic brain injury (TBI), particularly following decompressive craniectomy (DC) where there is an alteration of normal intracranial pressure (ICP) and cerebrospinal fluid (CSF) dynamics [1,2]. Rates of post-traumatic hydrocephalus vary between 0.7% and 29% in TBI patients and 10%-40% following DC [1,3]. Lumbar drain (LD) placement for continuous or intermittent CSF diversion is a common and effective method for confirming the diagnosis and treating hydrocephalus, including post-traumatic hydrocephalus. While LD is a relatively safe procedure, it still carries some risk to patients [4]. Among the most feared complications are intracranial hemorrhages, with a reported incidence of 0.6%-3% [4-6]. Hemorrhages have been noted in the subdural and subarachnoid spaces following LD placement, which was often thought secondary to CSF overdrainage [4,7-9]. Cases previously reported in the literature have focused on hemorrhages following LD for subarachnoid hemorrhage, aneurysm clipping, or intraoperative brain relaxation. We report here a case of brainstem hemorrhage following LD without overt CSF overdrainage in a TBI patient who had undergone decompressive craniectomy three months prior. To our knowledge, there is no report in the literature regarding brainstem hemorrhage following LD in a TBI patient. Given the propensity of TBI patients to develop post-traumatic hydrocephalus potentially requiring LD, it is important to recognize this potential catastrophic complication of a common procedure.

## Case presentation

**Figure 1:**
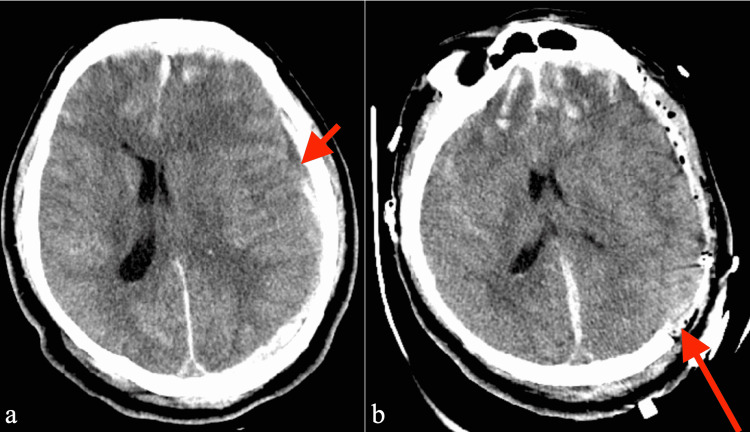
Preoperative (a) axial CT head showing acute/hyperacute left convexity subdural hematoma (short red arrow) causing left-to-right midline shift with compression of the left lateral ventricle. Bifrontal contusions were also demonstrated. Postoperative (b) axial CT head demonstrating large decompressive craniectomy (long red arrow) with the evacuation of subdural hematoma and improvement in midline shift.

A 32-year-old previously healthy male presented to a level 1 trauma center with severe TBI following a fall while intoxicated. On admission, the patient’s initial Glasgow Coma Scale (GCS) score was 3T, and he was intubated and was without a motor response to noxious stimuli. His pupils were symmetric and reactive to light bilaterally. Initial head computed tomography (CT) demonstrated a large 7-mm thick left acute subdural hematoma, nondepressed right skull fracture, bifrontal contusions, and a right cerebellar contusion (Figure 1, Panel a). The patient was taken emergently to the operating room for a left DC and subdural hematoma evacuation, expansile duraplasty, and placement of a parenchymal ICP monitor (Figure 1, Panel b). Postoperatively, the patient’s ICP was elevated, prompting the placement of an external ventricular drain (EVD) on postoperative day (POD) 1. The patient’s ICP and cerebral perfusion pressure (CPP) were thereafter well controlled, and the EVD was successfully removed after 12 days. Following an initial extubation on POD 11, the patient required additional reintubations on POD 14 and POD 23 due to respiratory distress and recurrent pneumonia, ultimately requiring a tracheostomy and gastrostomy tube placement one month after his injury. A CT angiogram of the head on POD 10 and magnetic resonance imaging (MRI) with and without contrast of the brain on POD 19 revealed no intracranial vascular abnormalities or nontraumatic mass lesions.

**Figure 2:**
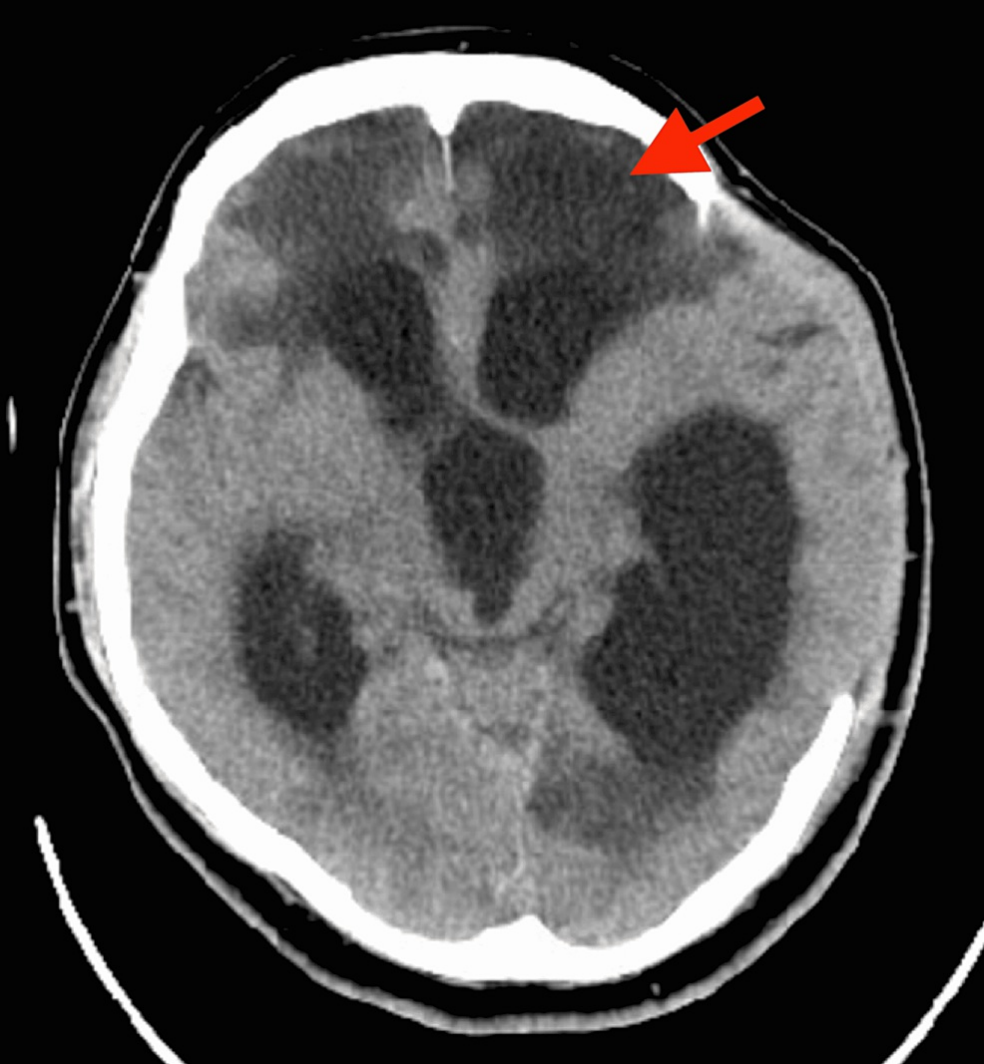
Axial CT head three months after injury, demonstrating ventriculomegaly with transependymal flow (red arrow) and mild herniation of brain parenchyma through craniectomy defect

After surgery and ventriculostomy, the patient’s neurological exam had improved to spontaneous eyes opening and following commands in the right hand, with a GCS of 11. He had been weaned off the ventilator but still required supplemental oxygen and was non-verbal. Three months after his head trauma, while still hospitalized awaiting long-term care placement, the patient was noted to have a gradual worsening of his exam, localizing with his right upper extremity, but no longer following commands. A repeat head CT was obtained demonstrating ventriculomegaly with transependymal flow consistent with presumed post-traumatic communicating hydrocephalus (Figure 2). Subsequently, an LD was successfully placed with a single dural puncture to confirm the diagnosis and to bridge the patient to definitive shunt placement. The opening pressure was 15 cm H_2_O. The LD was kept clamped and opened by the patient’s nurse to drain 15 mL CSF every two hours and then reclamped. The drain was a temporary bridge to a definitive ventriculoperitoneal shunt, which was being planned for within the week.

**Figure 3:**
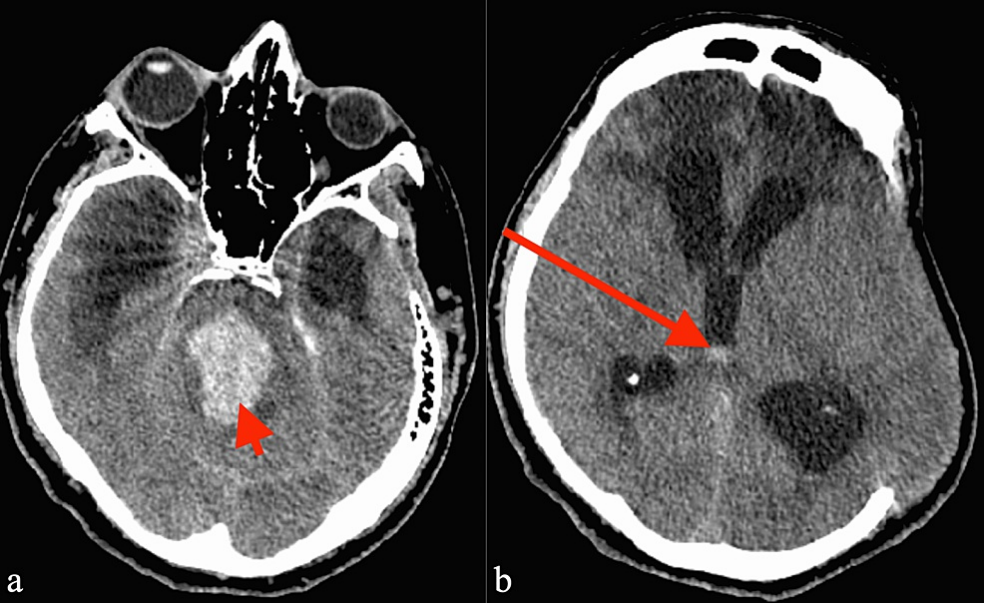
Axial CT head five days after lumbar drain demonstrating (a) catastrophic brainstem hemorrhage encompassing fourth ventricle (short red arrow) and (b) a mild decrease in the ventricle size and amount of brain parenchyma protruding through craniectomy skull defect, with intraventricular hemorrhage in the third ventricle (long red arrow)

Five days after LD placement, the patient developed an acute worsening of his neurological exam, with his GCS declining to 5, eyes open to noxious stimuli only, with no motor response to stimulation, and with bilaterally symmetric fixed and dilated pupils. Drain output had been 165 mL over the past 24 hours; however, the LD was noted to be sluggish, and only 5 mL CSF had been drained in the immediately preceding five-hour period. The CSF was noted to be newly blood-tinged. Additional CSF drainage was halted, and the LD was promptly removed. There was no indication of CSF leakage from around the catheter, nor any disconnection or dislodgement of the catheter/drain. A stat head CT was obtained, which demonstrated a catastrophic acute brainstem hemorrhage with extension into the fourth ventricle, without extra-axial hemorrhage or collections, or tonsillar herniation (Figure 3, Panel a). The lateral ventricles were decreased slightly in size from prior imaging, and there were no other hemorrhagic foci (Figure 3, Panel b). He was not hypertensive around the time of the hemorrhage. His only anticoagulation was subcutaneous heparin for venous thromboembolism prophylaxis. Further neurosurgical intervention was deemed futile. Supportive care was continued until the determination of brain death 11 days following LD placement.

## Discussion

LD has been associated with multiple forms of intracranial hemorrhage [4,7-9]. Most commonly, CSF overdrainage leads to subdural hemorrhage due to tearing of bridging veins. Small subarachnoid hemorrhage likely occurs through a similar mechanism but with smaller vessels. Remote cerebellar and parenchymal hemorrhages have also been described with several purported mechanisms. These include CSF hypovolemia leading to cerebellar sag and transient stretch and occlusion of cerebellar veins that then results in hemorrhagic venous infarction, deformation of small intraparenchymal arterioles leading to rupture, and direct injury to brain parenchyma from resting along the firm skull base for prolonged periods of time [10-12].

Brainstem hemorrhage following LD is a rare event that has been described only in a couple of case reports outside the neurosurgical literature [7,8]. These prior reports have been in the setting of overdrainage and CSF hypovolemia leading to transtentorial herniation [7,8]. The purported mechanism is downward transtentorial herniation of the brainstem relative to a more fixed basilar artery. Perforating pontine blood vessels then deform, stretch, and subsequently rupture, leading to the Duret hemorrhage. Brainstem blood vessels other than these basilar perforators can stretch and rupture by a similar mechanism, except without the accompanying transtentorial herniation.

In the absence of elevated ICP, transtentorial herniation can occur with excessive CSF drainage. Yuan et al. reported on a case of transtentorial herniation with Duret hemorrhage after rapid CSF overdrainage via LD [8]. Their patient previously underwent a craniectomy for a thalamic intraparenchymal hemorrhage and had 150 mL of CSF drained over 30 minutes [8]. Kakati et al. similarly described a fatal brainstem hemorrhage with transtentorial herniation following a large volume of CSF diversion via an intraoperative LD during aneurysm clipping [7].

An association between LD and transtentorial herniation (without brainstem hemorrhage) has been reported even in the absence of excessive drainage or elevated ICP. Bloch and Regli reported a patient with an LD placed after craniotomy for carotid aneurysm clipping [13]. The LD was not draining and was subsequently removed, yet the patient developed symptoms of transtentorial herniation three days later. There was radiographic transtentorial and tonsillar herniation without brainstem hemorrhage. The authors theorized that this may have been secondary to an occult lumbar CSF fistula, although there were other potential routes of CSF egress in the setting of craniotomy for aneurysm clipping and cranialization of the frontal sinus. Komotar et al. similarly found that 8.0% of aneurysmal subarachnoid hemorrhage patients undergoing craniotomy with preoperative or intraoperative LD demonstrated signs of transtentorial herniation attributable to CSF hypovolemia [9].

Our case is unique in that no apparent CSF overdrainage occurred, and there were no signs on neurologic examination or significant change in the fullness of the patient’s craniectomy scalp flap to suggest overdrainage prior to the patient’s acute clinical worsening. However, the temporal relationship between LD placement and the patient’s subsequent brainstem hemorrhage suggests causation. No other major changes had occurred with the patient’s care preceding his hemorrhage. He did not have a traumatic pseudoaneurysm with a prior computed tomography angiography (CTA) and MRI indicating no underlying vascular abnormality. Traumatic pseudoaneurysms are also more commonly present with subarachnoid hemorrhage and often within a week of trauma [14].

The exact mechanism by which our patient developed brainstem hemorrhage thus remains unclear. The clinical course and radiographic findings do not support any mechanism of transtentorial herniation being related to our patient’s brainstem hemorrhage. The lack of a bone flap could prevent the development of a substantial acute ICP gradient required to result in transtentorial or tonsillar herniation, thus downward herniation was not seen on the patient’s CT scan. 

Even still, it is possible that LD induced changes within the brainstem that led to the hemorrhage. While Komotar et al. did not report brainstem hemorrhages, they did note brainstem elongation in 30% of their patients [9]. Kim et al. also reported brainstem elongation due to CSF hypovolemia [15]. The brainstem appears to undergo a morphological change in response to CSF drainage, even in the absence of acute or sudden overdrainage.

With an absent bone flap and LD, our patient likely had a negative ICP. It is possible that the brainstem was vulnerable to the negative ICP environment coupled with increased CSF flow leading to the expansion of the brainstem with this expansion critical to the development of hemorrhage. This expansion theory is similar to the proposed Bernoulli theorem for syringomyelia [16]. Given that we did not obtain a CT scan in the interval between LD placement and the patient’s devastating brainstem hemorrhage, it is impossible to know whether brainstem elongation or expansion occurred prior to the hemorrhage.

## Conclusions

To our knowledge, our case of brainstem hemorrhage in a TBI patient following LD without apparent CSF overdrainage or transtentorial herniation has not been previously reported. Our case illustrates a grave potential complication of LD for the treatment of post-traumatic hydrocephalus. While intracranial hemorrhage resulting from LD is often related to CSF overdrainage, it is possible that the threshold for excessive drainage differs in a patient with altered CSF dynamics following DC compared to patients with more “normal” CSF flow dynamics. Additionally, TBI patients are often debilitated, and the signs and symptoms attributable to CSF hypovolemia or the beginning of transtentorial herniation may thus be confounded; this may render these patients more susceptible to progression to brainstem hemorrhage due to unrecognized overdrainage.

LD remains a viable and generally well-tolerated option for the diagnosis and treatment of communicating hydrocephalus, including post-traumatic hydrocephalus. However, neurosurgeons should remain aware of the potential for catastrophic brainstem hemorrhage following LD, particularly in the TBI population, despite the vigilance to prevent CSF overdrainage.
